# National Comparison of Ambulatory Physician Electronic Health Record Use Across Specialties

**DOI:** 10.1007/s11606-024-08930-4

**Published:** 2024-07-09

**Authors:** A Jay Holmgren, Christine A. Sinsky, Lisa Rotenstein, Nate C. Apathy

**Affiliations:** 1 University of California, San Francisco, CA USA; 2 American Medical Association, Chicago, IL USA; 3 University of Maryland, College Park, MD USA

## INTRODUCTION

Broad adoption of electronic health records (EHRs) has dramatically changed the nature of clinical care delivery. Physicians spend a significant amount of time working in the EHR,^1^ which has been associated with burnout.^2^ While early studies have characterized EHR use,^1^ they have been limited in their ability to normalize EHR time by clinical workload or differentiate work outside of work time between days with scheduled appointments and unscheduled days. Studies using organization-level data have shown EHR time increased significantly following the onset of the COVID-19 pandemic,^3^ yet the dynamics of that increase across specialties are unknown. It may be that increase was driven by previously low EHR time for clinicians, resulting in smaller differences across specialties, or EHR time could have increased evenly and the gap between specialties has remained static or increased. Establishing post-COVID onset EHR baselines is critical to tracking changes over time as well as evaluating the impact of policy efforts to reduce EHR burden. To address this, we use national EHR metadata to measure physician EHR time by specialty, the distribution of that time across EHR functions, and the proportion of time spent during compared to outside of clinic hours.

## METHODS

This cross-sectional study used de-identified data from all ambulatory physicians in the USA using an Epic Systems EHR from November 2021 through April 2022. Our sample included 200,081 unique physicians at 396 organizations from Epic’s Signal platform, which tracks “active” EHR time (any mouse activity or keystrokes) using a 5-s inactivity timeout.^4^ This study was deemed exempt by the UCSF Institutional Review Board.

We measured physician active time across four primary functions (documentation, chart review, orders, inbox) based on what function was being actively used at any given time as well as grouping all other EHR activity such as scheduling as “Other.” We then measured active EHR time across three categories of when that time occurred: during clinic hours, currently defined in Signal as beginning 30 min prior to the first appointment of the day through 30 min following the last appointment of the day; time outside of those scheduled hours on days with scheduled appointments, and time on unscheduled days. The latter two measures combined are referred to as “work outside of work.”^5^ All were normalized to 8-patient-care scheduled hours (PSH) to account for differences in clinical effort across physicians.^5^

## RESULTS

**Figure 1:**
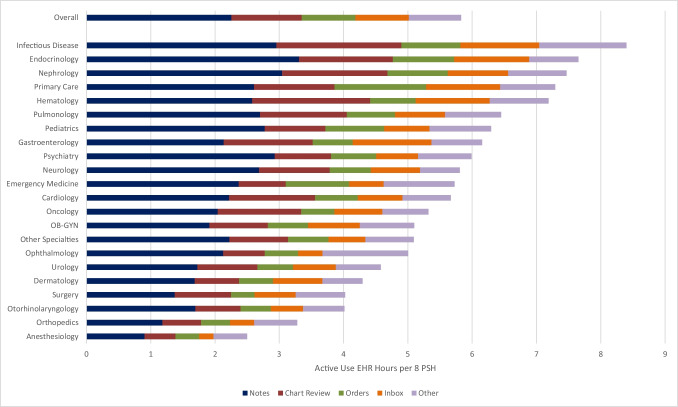
EHR time by function.

Physicians spent a mean of 5.8 h per 8 PSH actively working in the EHR (standard deviation (SD) 3.7). Physicians spent the most time in documentation with 2.3 h per 8 PSH (SD 1.8), followed by Chart Review (1.1 h per 8 PSH, SD 0.9), Orders (0.8 h, SD 0.7), Inbox (0.8 h, SD 0.8), with 0.8 h per 8 PSH across other EHR activities (SD 0.6) (Fig. 1). Infectious disease physicians had the highest EHR time (8.4 h per 8 PSH), followed by endocrinology (7.7 h), nephrology (7.5 h), and primary care (family and internal medicine) (7.3 h), while anesthesiology (2.5 h per 8 PSH) and orthopedics (3.3 h) had the lowest.

**Figure 2:**
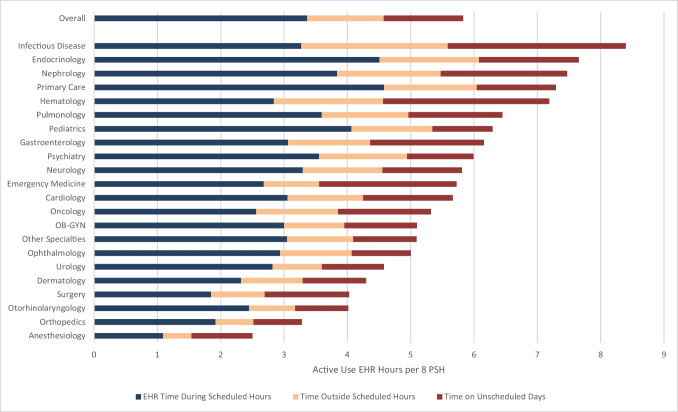
EHR time during clinic hours, time outside scheduled hours, and time on unscheduled days.

Physicians spent 3.4 h (57.8% of total time) of EHR time during clinic hours (SD 1.9), 1.2 h outside of clinic hours on days with scheduled appointments (20.7% of total time, SD 1.4), and 1.3 h on unscheduled days (21.5% of total time, SD 1.9) (Fig. 2). Infectious disease had the highest proportion of time outside clinic hours on scheduled days (27.6%), while anesthesiology had the highest proportion of time on unscheduled days (38.3%).

## DISCUSSION

Ambulatory physicians spend a significant amount of time actively using the EHR—nearly 6 h per 8 h of scheduled patient care. Further, a significant portion of EHR work takes place outside of clinic hours, which may be associated with decreased well-being and burnout.^2^ There is significant variation across specialties, with predominantly non-procedural specialties (e.g., primary care) registering greater EHR time relative to more procedural specialties (e.g., dermatology). This is especially true for Inbox time, where primary care physicians spend 50% more time compared to the overall average. Reducing EHR burden should be a critical priority for health system leaders, policymakers, technology vendors, and physicians.

Our study strengths include a large national sample, linked scheduling data to normalize across different clinical workloads, and detailed EHR metadata that tracks “active” EHR use. Limitations include data from a single EHR vendor,^6^ Signal’s definition of clinic hours may not match real-world scheduling practices, and our scheduling data is unable to account for protected time for EHR work. Future research should investigate how to reduce EHR burden and improve post-COVID era care processes.

## Declarations:

### Disclaimer

The opinions expressed in this article are those of the authors and should not be interpreted as American Medical Association policy.
